# Artificial Intelligence in Nuclear Medicine: Opportunities, Challenges, and Responsibilities Toward a Trustworthy Ecosystem

**DOI:** 10.2967/jnumed.121.263703

**Published:** 2023-02

**Authors:** Babak Saboury, Tyler Bradshaw, Ronald Boellaard, Irène Buvat, Joyita Dutta, Mathieu Hatt, Abhinav K. Jha, Quanzheng Li, Chi Liu, Helena McMeekin, Michael A. Morris, Peter J.H. Scott, Eliot Siegel, John J. Sunderland, Neeta Pandit-Taskar, Richard L. Wahl, Sven Zuehlsdorff, Arman Rahmim

**Affiliations:** 1 Department of Radiology and Imaging Sciences, Clinical Center, National Institutes of Health, Bethesda, Maryland;; 2 Department of Radiology, University of Wisconsin–Madison, Madison, Wisconsin;; 3 Department of Radiology and Nuclear Medicine, Cancer Centre Amsterdam, Amsterdam University Medical Centres, Amsterdam, The Netherlands;; 4 Institut Curie, Université PSL, INSERM, Université Paris–Saclay, Orsay, France;; 5 Department of Electrical and Computer Engineering, University of Massachusetts Lowell, Lowell, Massachusetts;; 6 LaTIM, INSERM, UMR 1101, University of Brest, Brest, France;; 7 Department of Biomedical Engineering and Mallinckrodt Institute of Radiology, Washington University, St. Louis, Missouri;; 8 Department of Radiology, Massachusetts General Hospital and Harvard Medical School, Boston, Massachusetts;; 9 Department of Radiology and Biomedical Imaging, Yale University, New Haven, Connecticut;; 10 Department of Clinical Physics, Barts Health NHS Trust, London, United Kingdom;; 11 Department of Radiology, University of Michigan Medical School, Ann Arbor, Michigan;; 12 Department of Radiology and Nuclear Medicine, University of Maryland Medical Center, Baltimore, Maryland;; 13 Departments of Radiology and Physics, University of Iowa, Iowa City, Iowa;; 14 Department of Radiology, Memorial Sloan Kettering Cancer Center, New York, New York;; 15 Mallinckrodt Institute of Radiology, Washington University, St. Louis, Missouri;; 16 Siemens Medical Solutions USA, Inc., Hoffman Estates, Illinois; and; 17 Departments of Radiology and Physics, University of British Columbia, Vancouver, British Columbia, Canada

**Keywords:** artificial intelligence, trustworthy, nuclear medicine, ecosystem

## Abstract

Trustworthiness is a core tenet of medicine. The patient–physician relationship is evolving from a dyad to a broader ecosystem of health care. With the emergence of artificial intelligence (AI) in medicine, the elements of trust must be revisited. We envision a road map for the establishment of trustworthy AI ecosystems in nuclear medicine. In this report, AI is contextualized in the history of technologic revolutions. Opportunities for AI applications in nuclear medicine related to diagnosis, therapy, and workflow efficiency, as well as emerging challenges and critical responsibilities, are discussed. Establishing and maintaining leadership in AI require a concerted effort to promote the rational and safe deployment of this innovative technology by engaging patients, nuclear medicine physicians, scientists, technologists, and referring providers, among other stakeholders, while protecting our patients and society. This strategic plan was prepared by the AI task force of the Society of Nuclear Medicine and Molecular Imaging.

Medicine uses science, practical wisdom, and the best available tools in the art of compassionate care. The necessity of dealing with maladies has motivated physicians to incorporate inventions into medical practice to decrease or eliminate patient suffering. During the past two centuries, along with technologic revolutions, new medical devices have become the standard of care, from the stethoscope and electrocardiogram to cross-sectional imaging (Fig. 1). The stethoscope, which arose out of the first industrial revolution, is so pervasive that it has become the symbol of health-care professionals today. Compared with other medical equipment, it has the highest positive impact on the perceived trustworthiness of the practitioner seen with it (1).

**FIGURE 1.**
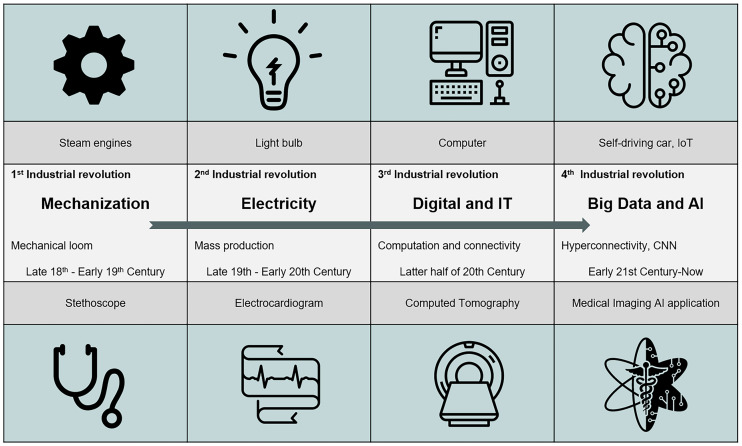
New technologies in medicine have coincided with each phase of industrial revolution. First industrial revolution was mechanization, with mechanical loom invented in 1784. The stethoscope was invented by René Laennec in 1816 and improved by Arthur Leared (1851) and George Philip Cammann (1852). Second industrial revolution was driven by advent of electricity, with the commercial light bulb (patented by Thomas Edison in 1879), telegram, and modern factory production line. Electrocardiogram was invented by Augustus Waller in 1887 by projecting the heartbeat captured by Lippmann capillary electrometer onto photographic plate, allowing heartbeat to be recorded in real time. Willem Einthoven (1895) assigned letters P, Q, R, S, and T to the theoretic waveform. Third industrial revolution, known as digital revolution, brought computing technology and refined it to personal computer. In 1960s, Kuhl and Edwards developed cross-sectional CT and implemented this in the SPECT scanner, which was later applied to CT scanner by Sir Godfrey Hounsfield and Allan Cormack in 1972. Fourth industrial revolution is that of modern day, with big data, hyperconnectivity, and neural networks, resulting in ability to propel self-driving cars and development of AI in nuclear medicine. CNN = convolutional neural network; IoT = Internet of things.

Nuclear medicine has always embraced the progress of technology. With the emergence of AI, we will again be poised to experience a modern renaissance, similar to the one experienced after David Kuhl’s and Roy Edwards’ groundbreaking work in the 1960s. By applying the concepts of radon transform through newly available computing technology, they introduced volumetric cross-sectional medical imaging with SPECT, which was subsequently followed by the development of x-ray–based CT and PET (2).

The past decades have seen tremendous advances in information technology and in its integration into the practice of medicine. The application of artificial intelligence (AI) to medicine represents the actualization of a new era. Such transformative technologies can affect all facets of society, yielding advances in space exploration, defense, energy, industrial processes, and finance; and even in cartography, transportation, and food service, among others.

The addition of AI into clinical practice in nuclear medicine poses opportunities and challenges. The full benefits of this new technology will continuously evolve. It is important to recognize that the nuclear medicine community must be actively involved to ensure safe and effective implementation. Establishing and maintaining AI leadership in the realm of nuclear medicine requires a comprehensive strategy to promote the application of innovative technology while protecting our patients and society, executing our professional and ethical obligations, and promoting our values. A potential advantage of deploying AI techniques is that nuclear medicine methodologies may become more widely available, increasing the access of patients to high-quality nuclear medicine procedures.

Nuclear medicine professional societies such as the Society of Nuclear Medicine and Molecular Imaging (SNMMI) and others provide leadership to ensure that we recognize the benefits of technologic advances in a manner consistent with our core values, medical ethics, and society’s best interests. In July 2020, the SNMMI formed an AI task force by bringing together experts in nuclear medicine and AI, including physicists, computational imaging scientists, physicians, statisticians, and representatives from industry and regulatory agencies. This article serves as both a strategic plan and a summary of the deliberations of the SNMMI AI task force over the past year in conjunction with other focused topics, including best practices for development (3) and evaluation (4) (Table 1).

**NOTEWORTHY**

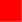
 An appropriate AI ecosystem can contribute to enhancing the trustworthiness of AI tools throughout their life cycle through close collaboration among stakeholders.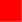 A trustworthy medical AI system depends on the trustworthiness of the AI system itself, as well as the trustworthiness of all people and processes that are part of the system’s life cycle.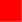 By encouraging the establishment of trustworthy AI in nuclear medicine, SNMMI aims to decrease health disparity, increase health system efficiency, and contribute to the improved overall health of society using AI applications in the practice of nuclear medicine.

**TABLE 1.** Opportunities and Challenges Ahead for Nuclear Medicine Toward Achieving Trustworthy AI

Category	Domain	Subdomain
Opportunities	Diagnostic imaging	Emerging nuclear imaging approaches
	RPTs	AI-driven theranostic drug discovery and labeling
		Precision dosimetry
		Predictive dosimetry and digital twins
	Clinical workflow: increasing throughput while maintaining excellence	
Challenges	Development of AI applications/medical devices	Data
		Optimal network architecture
		Measurement and communication of uncertainty
		Clinically impactful use cases
		Team science
	Evaluation (verification of performance)	Performance profiling through task-based evaluations
		Guidelines for validation
		Multicenter clinical trial network
	Ethical, regulatory, and legal ambiguities	Ethical aspects
		Regulatory and legal aspects
	Implementation of clinical AI solutions and postimplementation monitoring	AI platform
		Barriers of dissemination and implementation of AI technology in medicine
		Postdeployment: change management and performance
	Trust and trustworthiness	

## OPPORTUNITIES

### Quantitative Imaging and Process Improvement

Nuclear medicine is evolving toward even better image quality and more accurate and precise quantification in the precision medicine era, most recently in the paradigm of theranostics.

### Diagnostic Imaging

**FIGURE 2.**
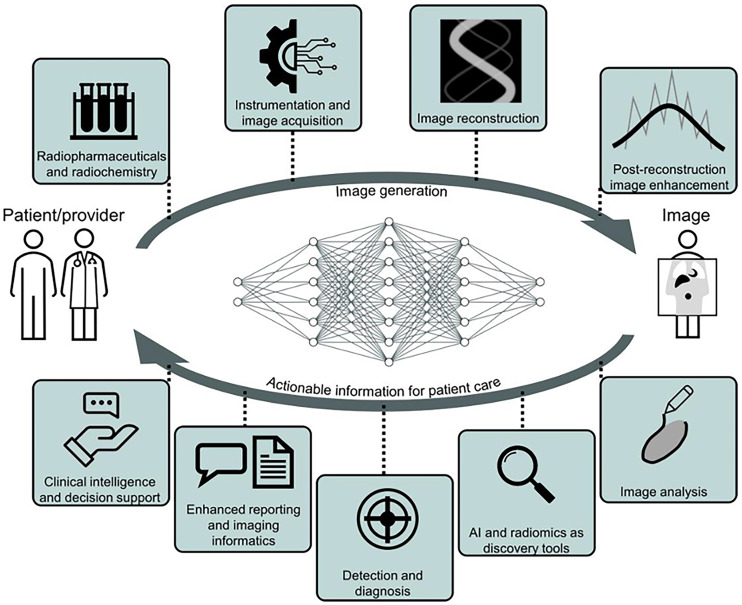
From patient to image creation and back to physician, there are opportunities for AI systems to act at nearly any step in medical imaging pipeline to improve our ability to care for patients and understand disease (3).

AI techniques in the patient-to-image subdomain improve acquisition, and models in the image-to-patient subdomain improve decision making for interventions on patients (Fig. 2) (3).

Image generation considerations are elaborated in the supplemental section “Opportunities,” part A (supplemental materials are available at http://jnm.snmjournals.org (5–40)); however, examples include improved image reconstruction from raw data (list-mode, sinogram); data corrections including for attenuation, scatter, and motion; and postreconstruction image enhancement, among others (41–43). These enhancements could impact PET and SPECT in clinical use today. Multiple–time-point acquisitions and PET/MRI may see improved feasibility.

Specific opportunities in image analysis are elaborated in the supplemental section “Opportunities,” part B. A few examples include image registration, organ and lesion segmentation, biomarker measurements and multiomics integration, and kinetic modeling (44).

Opportunities for clinical use of AI in nuclear medicine practice were extensively reviewed recently, including brain imaging (45), head and neck imaging (46), lung imaging (47), cardiac imaging (48*,*^49^), vascular imaging (49*,*^50^), bone imaging (51), prostate imaging (52), and imaging of lymphoma (53). Neuroendocrine tumors, other cancers (including gastrointestinal, pancreatic, hepatobiliary, sarcoma, and hereditary), infection, and inflammation are some examples of additional areas requiring further consideration.

### Emerging Nuclear Imaging Approaches

New developments are also emerging such as total-body PET (54), which presents unique data and computational challenges. Another potential use of AI is to separate multichannel data from single-session multiisotope dynamic PET imaging. This pragmatic advancement could be valuable to extract greater phenotyping information in the evaluation of tumor heterogeneity (55).

### Radiopharmaceutical Therapies (RPTs)

There are several areas in which AI is expected to significantly impact RPTs.

#### AI-Driven Theranostic Drug Discovery and Labeling

The use of AI for molecular discovery has been explored to select the most promising leads to design suitable theranostics for the target in question. For example, machine learning models could be trained using parameters from past theranostic successes and failures (e.g., partition coefficient, dissociation constant, and binding potential) to establish which best predict a given outcome (e.g., specific binding, blood–brain barrier penetration, and tumor-to-muscle ratio). New AI approaches are revolutionizing our understanding of protein–ligand interactions (56). New hit molecules (e.g., from the literature or high-throughput screens) can then serve as the test set in such AI models to speed up hit-to-lead optimization. Subsequently, with lead molecules identified, AI could also predict optimal labeling precursors and synthesis routes to facilitate fast and efficient development of theranostic agents (57*,*^58^). By defining parameters from existing synthetic datasets (e.g., solvents, additives, functional groups, and nuclear magnetic resonance shifts), models can be trained to predict radiochemical yield for a given substrate using different precursors and radiosynthetic methods. Subjecting new lead candidates as test sets in the models will enable rapid identification of appropriate precursors and labeling strategies for new theranostics, minimizing resource-intensive manual synthetic development.

#### Precision Dosimetry

**FIGURE 3.**
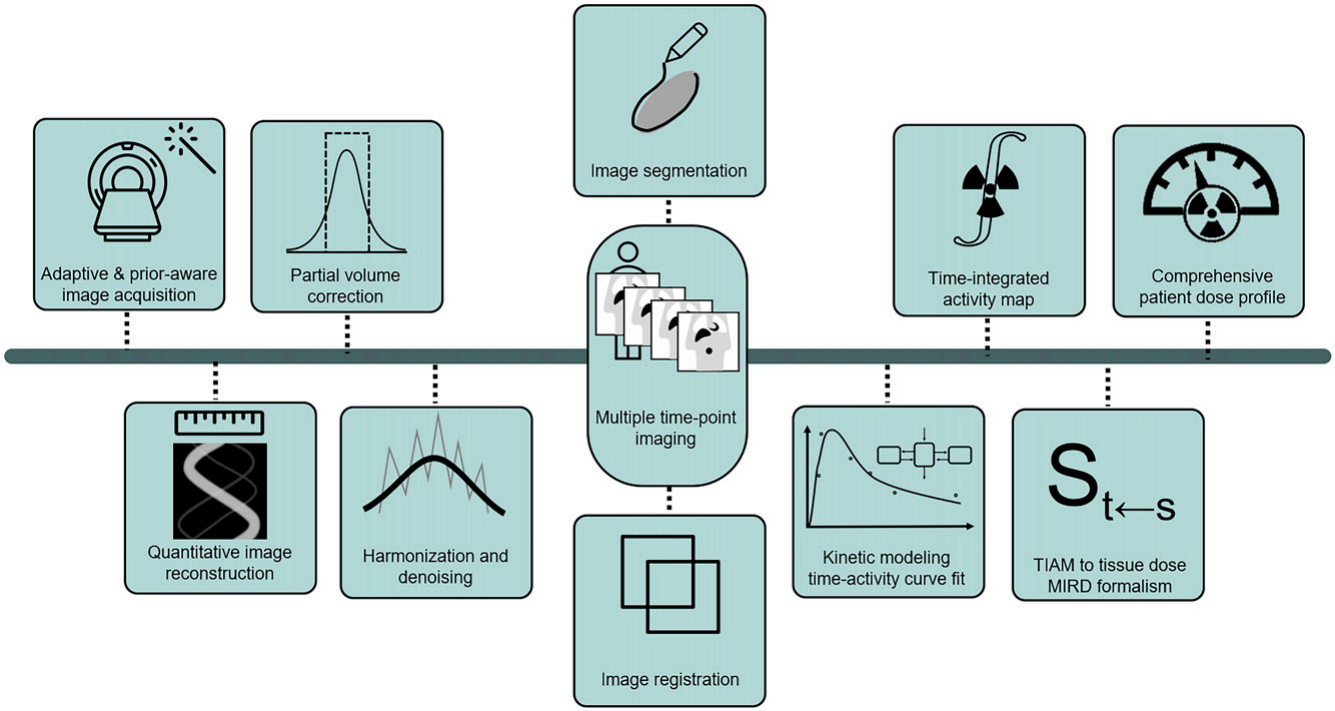
Dosimetry as major frontier supported by AI toward personalization of therapy: various contributions by AI to image acquisition, generation, and processing, followed by automated dose calculations, can enable routine deployment and clinical decision support. TIAM = Time Integrated Activity Map.

The field of radiopharmaceutical dosimetry is progressing rapidly. After administration of radiopharmaceuticals, dynamic and complex pharmacokinetics results in time-variable biodistribution. Interaction of ionizing particles arising from the injected agent with the target and normal tissue results in energy deposition. Quantification of this deposited energy and its biologic effect is the essence of dosimetry, with opportunities to link the deposited energy to its biologic effect on diseased and normal tissues (Fig. 3).

In dosimetry, SPECT serves as a posttreatment quantitative measuring device. One challenge is the difficulty for patients to remain flat and motionless on the scanning table for the required time. AI-based image reconstruction or enhancement methods can reduce the required SPECT scanning time for patients while maintaining or enhancing the accuracy of quantification (59) and enable attenuation correction in SPECT (60).

Multiple steps in dosimetry potentially can be enhanced by AI methods, including multimodality and multiple–time-point image registration, segmentation of organs and tumors, time–activity curve fitting, time-integrated activity estimation, conversion of time-integrated activity into absorbed dose, linking macroscale dosimetry to microscale dosimetry, and arriving at comprehensive patient dose profiling (61).

#### Predictive Dosimetry and Digital Twins

Existing models can perform dosimetry before (e.g., ^131^I-metaiodobenzylguanidine) or after treatment. Personalized RPTs require predictive dosimetry for optimal dose prescription in which AI can play a role. Pretherapy (static or dynamic) PET scans could model radiopharmaceutical pharmacokinetics and absorbed doses in tumors and normal organs. Furthermore, it is possible to additionally use intratherapy scans (e.g., single–time-point SPECT in the first cycle of RPTs) to better anticipate and adjust doses in subsequent cycles.

Overall, a vision of the future involves accurate and rapid evaluation of different RPT approaches (e.g., varying the injected radioactivity dose and rate, site of injection, and injection interval and coupling with other therapies) using the concept of the theranostic digital twin. The theranostic digital twin can aid nuclear medicine physicians in complex decision-making processes. It enables experimentation (in the digital world) with different treatment scenarios, thus optimizing delivered therapies.

The opportunities discussed in the RPT section above are further described in the supplemental section “Opportunities,” part C.

### Clinical Workflow: Increase Throughput While Maintaining Excellence

AI may impact operations in nuclear medicine, such as patient scheduling and resource use (62), predictive maintenance of devices to minimize unexpected downtimes, monitoring of quality control measurement results to discover hidden patterns and indicate potential for improvement, and monitoring of the performance of devices in real time to capture errors and detect aberrancies (62*,*^63^). These processes will make the practice of nuclear medicine safer, more reliable, and more valuable.

Triage of urgent findings and augmentation of time-consuming tasks could improve the report turnaround time for the most critical cases and increase the efficiency of nuclear medicine physicians, allowing them to more effectively care for patients. It is important to ensure that AI systems in nuclear medicine are sustainable through developing new current procedural terminology codes and assigning appropriate relative value units for the technical and professional components. It is also possible that increased efficiencies in interpretation (more cases read accurately per unit time) may allow AI to be deployed into clinical workflows in an overall cost-effective manner.

## AI ECOSYSTEM

### Actualization of Opportunities and Contextualization of Challenges

Although early nuclear medicine AI systems are already emerging, many opportunities remain in which the continuous propagation of AI technology could augment our precision patient care and practice efficiencies. The environment in which AI development, evaluation, implementation, and dissemination occurs needs a sustainable ecosystem to enable progress, while appropriately mitigating concerns of stakeholders.

**FIGURE 4.**
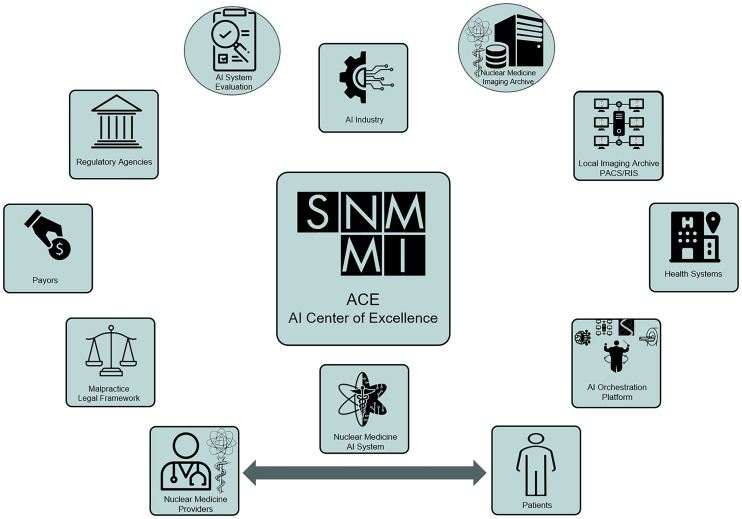
AI ecosystem is a complex environment in which AI system development occurs. The ecosystem connects stakeholders from industry to regulatory agencies, physicians, patients, health systems, and payers. Proposed SNMMI AI Center of Excellence can serve as an honest broker to empower the AI ecosystem from a neutral standpoint with focus on solutions. ACE = SNMMI AI Center of Excellence; RIS = radiology information system.

The total life cycle of AI systems, from concept to appropriation of training data, model development and prototyping, production testing, validation and evaluation, implementation and deployment, and postdeployment surveillance, occurs within a framework that we call the AI ecosystem (Fig. 4). An appropriate AI ecosystem can contribute to enhancing the trustworthiness of AI tools throughout their life cycle through close collaboration among stakeholders.

## CHALLENGES FOR DEVELOPMENT, VALIDATION, DEPLOYMENT, AND IMPLEMENTATION

### Development of AI Applications and Medical Devices

Five challenges that should be addressed include availability of curated data, optimization of network architecture, measurement and communication of uncertainty, identification of clinically impactful use cases, and improvements in team science approaches (supplemental section “Development Challenges”).

### Evaluation (Verification of Performance)

Theories on appropriate evaluation of AI software are a broad and active area of current investigation. Establishing clear and consistent guidelines for performance profiling remains challenging. Most current verification studies evaluate AI methods on the basis of metrics that are agnostic to performance on clinical tasks (64). Although such evaluation may help demonstrate promise, there is an important need for further testing on specific clinical tasks before the algorithms can be implemented. Failure-mode profiling is among the most important challenges (supplemental section “Evaluation Challenges”).

### Ethical, Regulatory, and Legal Ambiguities

Major ethical concerns include informed consent for data use, replication of historical bias and unfairness embedded in training data, unintended consequences of AI device agency, the inherent opaqueness of some algorithms, concerns about the impact of AI on health-care disparities, and trustworthiness (supplemental section “Ethical, Regulatory, and Legal Ambiguities”). AI in nuclear medicine has limited legal precedent (65).

### Implementation of Clinical AI Solutions and Postdeployment Monitoring

The lack of an AI platform integrating AI applications in the nuclear medicine workflow is among the most critical challenges of implementation (66). Barriers of dissemination can be categorized at the individual level (health-care providers), at the institutional level (organization culture), and at the societal level (67). Deployment is not the end of the implementation process (supplemental section “Implementation of Clinical AI Solutions and Post-Deployment Monitoring”).

## TRUST AND TRUSTWORTHINESS

In medicine, trust is the essence, not a pleasance.

Successful solutions to the above-mentioned challenges are necessary but not sufficient for the sustainability of AI ecosystems in medicine. Well-developed and validated AI devices with supportive regulatory context, appropriate reimbursement, and successful primary implementation may still fail if physicians, patients, and society lose trust because of lack of transparency and other critical elements of trustworthiness such as perceived inattention to health disparity or racial injustice. In a recent survey, Martinho et al. (68) found significant perceived mistrust among health-care providers with regard to AI systems and the AI industry while realizing the importance and benefits of this new technology. Responders also emphasized the importance of ethical use, and the need for physician-in-the-loop interactions with AI systems, among the other factors. There is a need for a comprehensive analysis of the AI ecosystem to define and clarify the core elements of trustworthiness in order to realize the benefits of AI in clinical practice.

## RESPONSIBILITIES: TOWARD TRUSTWORTHY AI

When the safety, well-being, and rights of our patients are at stake, SNMMI should be committed to support principles that are future-proof and innovation-friendly.

The willingness of physicians and patients to depend on a specific tool in a risky situation is the measure of the trustworthiness of that tool (69). In the case of AI systems, that willingness is based on a set of specific beliefs about the reliability, predictability, and robustness of the tool, as well as the integrity, competency, and benevolence of the people or processes involved in the AI system’s life cycle (development, evaluation/validation, deployment/implementation, and use).

**FIGURE 5.**
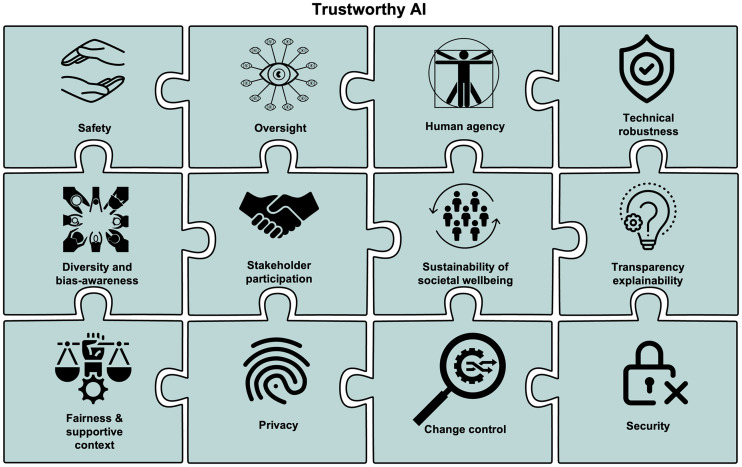
Twelve core concepts critical to trustworthy AI ecosystems.

A trustworthy medical AI system depends on the trustworthiness of the AI system itself, as well as the trustworthiness of all people and processes that are part of the system’s life cycle (Fig. 5).

Trustworthy medical AI systems require a societal and professional commitment to the ethical AI framework, which includes 4 principles rooted in the fundamentals of medical ethics: respect for patients’ and physicians’ autonomy, prevention of harm, beneficence to maximize the well-being of patients and society, and fairness. These principles should be observed in various phases of the AI system life cycle.

In what follows, we outline 12 key elements that need to be consistently present in AI systems.

### 12 Key Elements of Trustworthy AI Systems

#### Human Agency

AI systems should empower physicians and patients, allowing them to make better-informed decisions and foster their autonomy (70). Effects of the AI algorithms on human independence should be considered. It should be clear to patients and physicians the extent to which AI is involved in patient care and the extent of physician oversight. There must be checks to avoid automation bias, which is the propensity of humans to value and overly rely on observations and analyses from computers over those of human beings (71).

#### Oversight

There must be sufficient oversight of AI decision making, which can be achieved through human-in-the-loop and human-in-command approaches (72). AI systems that are involved in higher-risk tasks (e.g., those that drive clinical management and diagnose or treat disease) must be closely monitored through postmarket surveillance by independent professional credentialing organizations analogous to certification and recertification of medical professionals. Peer review processes in practices can be adapted to consider the combined physician–AI decision-making process.

#### Technical Robustness

AI systems must perform in a dependable manner (sufficient accuracy, reliability, and reproducibility) (73). This performance should be resilient to the breadth of clinical circumstances related to their prescribed use (generalizability). The AI tool should explicitly convey a degree of certainty about its output (confidence score) and have a mechanism in place to monitor the accuracy of outputs as part of a continuous quality assurance program. Failure modes of the algorithm should be well-characterized, documented, and understood by users.

#### Safety and Accountability

According to the concepts of safety-critical systems (74), AI systems should prioritize safety above other design considerations (e.g., potential gains in efficiency, economics, or performance). When adverse events occur, mechanisms should be in place for ensuring accountability and redress. Vendors must be accountable for the claims made of their AI systems. Physicians must be accountable for the way in which AI systems are implemented and used in the care of patients. The ability to independently audit the root cause of a failure in an AI system is important. Protection must be provided for individuals or groups reporting legitimate concerns in accordance with the principles of risk management.

#### Security and Data Governance

AI systems must include mechanisms to minimize harm, as well as to prevent it whenever possible. They must comply with all required cybersecurity standards. There should be an assessment of vulnerabilities such as data poisoning, model evasion, and model inversion. Assurances should be made to mitigate potential vulnerabilities and avoid misuse, inappropriate use, or malicious use (such as a deep fake) (75).

#### Predetermined Change Control Plan

AI tools can be highly iterative and adaptive, which may lead to rapid continual product improvement. The plan should include types of anticipated modifications (software-as-a-medical-device prespecifications). There must be a clear and well-documented methodology (algorithm change protocol) to evaluate the robustness and safety of the updated AI system. The algorithm change protocol should include guidelines for data management, retraining, performance evaluation, and update procedures. Vendors should maintain a culture of quality and organizational excellence.

#### Diversity, Bias Awareness, Nondiscrimination, and Fairness

AI systems can be affected by input data maladies (incomplete data, inadvertent historically biased data), algorithm design insufficiencies, or suboptimal performance assessment or monitoring strategies. These issues may result in biases leading to unintended prejudice and cause harm to patients. Discriminatory bias should be removed from AI systems in the development phase when possible (67).

AI system performance should be evaluated in a wide spectrum of diseases and in patients with a particular condition regardless of extraneous personal characteristics. No particular group of patients should be systematically excluded from AI device development. Patients who are underrepresented or have rare diseases should not be excluded from AI system development or evaluation—though such datasets will be sparse and most likely could be used in the evaluation of AI methods developed only in larger populations (for generalizability). Appropriate validation testing on standardized sets that incorporate patient diversity, including rare or unusual presentations of disease, are critical to evaluate the presence of bias in results regardless of the training data used (76).

AI systems should be user-centric and developed with an awareness of the practical limitations of the physician work environment. Accessibility features should be provided to those individuals with disabilities to the extent necessary according to universal design principles.

#### Stakeholder Participation

Throughout the life cycle of an AI system, all stakeholders who may directly or indirectly be affected should actively participate to help, advise, and oversee the developers and industry. Participation of patients, physicians, and all relevant providers, health-care systems, payors, regulatory agencies, and professional societies is imperative. This inclusive and transparent engagement is essential for a trustworthy AI ecosystem. Regular clinical feedback is needed to establish longer-term mechanisms for active engagement.

#### Transparency and Explainability

Vendors should openly communicate how an AI system is validated for the labeled claim (purpose, criteria, and limitations) by describing the clinical task for which the algorithm was evaluated; the composition of the patient population used for validation; the image acquisition, reconstruction, and analysis protocols; and the figure of merit used for the evaluation (4*,*^73^). There must be appropriate training material and disclaimers for health-care professionals on how to adequately use the system. It should be clear which information is communicated from the AI system and which information is communicated by a health-care professional. AI systems should incorporate mechanisms to log and review which data, AI model, or rules were used to generate certain outputs (auditability and traceability). The effect of the input data on the AI system’s output should be conveyed in a manner whereby their relationship can be understood by physicians and, ideally, patients (explainability) in order to allow a mechanism to critically evaluate and contest the AI system outputs. For diagnostic applications, the AI system should communicate the degree of confidence (uncertainty) together with its decision. To the extent possible, in high-stakes tasks the use of black box AI systems without proper emphasis on transparency should be avoided (77).

#### Sustainability of Societal Well-Being

It is important to acknowledge that exposure to AI could negatively impact social relationships and attachment within the health-care system (social agency) (78). AI systems should be implemented in a manner that enhances the physician–patient relationship. AI systems should not interfere with human deliberation or deteriorate social interactions. The societal and environmental impact of an AI tool should be carefully considered to ensure sustainability. Health-care workers who are impacted by the implementation of AI systems should be given an opportunity to provide feedback and contribute to its implementation plan. Professional societies and training programs should take steps to ensure that AI systems do not result in deskilling of professionals, such as by providing opportunities for reskilling and upskilling. A new set of skills, including physician oversight and interaction with AI tools, will evolve and must be refined.

#### Privacy

AI systems should have appropriate processes in place to maintain the security and privacy of patient data. The amount of personal data used should be minimized (data minimization). There should be a statement on measures used to achieve privacy by design, such as encryption, pseudoanonymization, aggregation, and anonymization. Systems should be aligned with standards and protocols for data management and governance.

#### Fairness and Supportive Context of Implementation

Early development efforts can pose more risk to developers and consumers. To address liability concerns, there have been successful programs in other industries to encourage adoption of new technology and support consumer protection, such as for vaccines and autonomous vehicles (65).

## STRATEGIES FOR SUCCESS

### Part 1: SNMMI Initiatives

In July 2022, SNMMI created an AI task force to strategically assess the emergence of AI in nuclear medicine (supplemental section “SNMMI Initiatives”). An area of important focus was to designate working groups, such as the AI and dosimetry working group for predictive dosimetry and treatment planning.

### Part 2: SNMMI Action Plan

The AI task force recommends the establishment of an SNMMI AI Center of Excellence to facilitate a sustainable AI ecosystem (supplemental section “SNMMI Action Plan”). A nuclear medicine imaging archive will address the need for meaningful data access. A coalition on trustworthy AI in medicine and society will address the need for an AI bill of rights (79).

### Part 3: SNMMI Recommendations

Recommendations for the future are also provided in the supplemental section “SNMMI Recommendations.”

## CONCLUSION

There are immense and exciting opportunities for AI to benefit the practice of nuclear medicine. Meanwhile, there are challenges that must and can be addressed head-on. As current challenges are addressed and new AI solutions emerge, SNMMI and the nuclear medicine community have the responsibility to ensure the trustworthiness of these tools in the care of patients.

We can all benefit from efforts to ensure fairness, inclusion, and lack of bias in the entire life cycle of AI algorithms in different settings.

There are 3 levels of facilitation that can support and enable the appropriate environment for trustworthy AI. First, our community must establish guidelines, such as those referenced in this article, to promote the natural development of trustworthy AI. Second, we can facilitate trustworthy AI through an SNMMI AI Center of Excellence. Third, we can make trustworthy AI occur through active engagement and communicative actions.

By encouraging the establishment of trustworthy AI in nuclear medicine, SNMMI aims to decrease health disparity, increase health system efficiency, and contribute to the improved overall health of society using AI applications in the practice of nuclear medicine.

## DISCLOSURE

The views expressed in this article are those of the authors and do not necessarily reflect the views of the U.S. government, nor do they reflect any official recommendation or endorsement of the National Institutes of Health. Helena McMeekin is a part-time employee of Hermes Medical Solutions, Inc. Sven Zuehlsdorff is a full-time employee of Siemens Medical Solutions, Inc. No other potential conflict of interest relevant to this article was reported.

## References

[1] Jiwa M Millett S Meng X Hewitt VM. Impact of the presence of medical equipment in images on viewers’ perceptions of the trustworthiness of an individual on-screen. J Med Internet Res. 2012;14:e100. 22782078 10.2196/jmir.1986 PMC3409609

[2] Dunnick NR David E. Kuhl, MD. Radiology. 2017;285:1065. 29155630 10.1148/radiol.2017174024

[3] Bradshaw TJ Boellaard R Dutta J. Nuclear medicine and artificial intelligence: best practices for algorithm development. J Nucl Med. 2022;63:500–510. 34740952 10.2967/jnumed.121.262567 PMC10949110

[4] Jha AK Bradshaw TJ Buvat I. Nuclear medicine and artificial intelligence: best practices for evaluation (the RELAINCE guidelines). J Nucl Med. 2022;63:1288–1299. 35618476 10.2967/jnumed.121.263239 PMC9454473

[5] Saboury B Rahmim A Siegel E. PET and AI trajectories finally coming into alignment. PET Clin. 2021;16:15–16. 34537135 10.1016/j.cpet.2021.07.003

[6] Saboury B Rahmim A Siegel E. Taming the complexity: using artificial intelligence in a cross-disciplinary innovative platform to redefine molecular imaging and radiopharmaceutical therapy. PET Clin. 2022;17:17–19. 10.1016/j.cpet.2021.11.002 34809876

[7] Reader AJ Schramm G. Artificial intelligence for PET image reconstruction. J Nucl Med. 2021;62:1330–1333. 34244357 10.2967/jnumed.121.262303

[8] Shiri I Ghafarian P Geramifar P. Direct attenuation correction of brain PET images using only emission data via a deep convolutional encoder-decoder (Deep-DAC). Eur Radiol. 2019;29:6867–6879. 31227879 10.1007/s00330-019-06229-1

[9] Yu Z Rahman MA Schindler T Laforest R Jha AK. A physics and learning-based transmission-less attenuation compensation method for SPECT. Proc SPIE Int Soc Opt Eng. 2021:11595. 10.1117/12.2582350 PMC8513502 34658480

[10] Shiri I Arabi H Geramifar P. Deep-JASC: joint attenuation and scatter correction in whole-body ^18^F-FDG PET using a deep residual network. Eur J Nucl Med Mol Imaging. 2020;47:2533–2548. 32415552 10.1007/s00259-020-04852-5

[11] Liu F Jang H Kijowski R Zhao G Bradshaw T McMillan AB. A deep learning approach for ^18^F-FDG PET attenuation correction. EJNMMI Phys. 2018;5:24. 30417316 10.1186/s40658-018-0225-8 PMC6230542

[12] Van Hemmen H Massa H Hurley S Cho S Bradshaw T McMillan A. A deep learning-based approach for direct whole-body PET attenuation correction [abstract]. J Nucl Med. 2019;60(suppl 1):569.

[13] Rahman A Zhu Y Clarkson E Kupinski MA Frey EC Jha AK. Fisher information analysis of list-mode SPECT emission data for joint estimation of activity and attenuation distribution. Inverse Probl. 2020;36:084002. 33071423 10.1088/1361-6420/ab958b PMC7561050

[14] Qian H Rui X Ahn S. Deep learning models for PET scatter estimations. In: *2017 IEEE Nuclear Science Symposium and Medical Imaging Conference (NSS/MIC). IEEE*; 2017:1–5.

[15] Arabi H Bortolin K Ginovart N Garibotto V Zaidi H. Deep learning-guided joint attenuation and scatter correction in multitracer neuroimaging studies. Hum Brain Mapp. 2020;41:3667–3679. 32436261 10.1002/hbm.25039 PMC7416024

[16] Sanaat A Arabi H Mainta I Garibotto V Zaidi H. Projection space implementation of deep learning-guided low-dose brain PET imaging improves performance over implementation in image space. J Nucl Med. 2020;61:1388–1396. 31924718 10.2967/jnumed.119.239327 PMC7456177

[17] Yu Z Rahman MA Schindler T. AI-based methods for nuclear-medicine imaging: need for objective task-specific evaluation [abstract]. J Nucl Med. 2020;61(suppl 1):575.

[18] Fu Y Lei Y Wang T Curran WJ Liu T Yang X. Deep learning in medical image registration: a review. Phys Med Biol. 2020;65:20TR01. 10.1088/1361-6560/ab843e PMC7759388 32217829

[19] Yousefirizi F. Pierre Decazes, Amyar A, Ruan S, Saboury B, Rahmim A. AI-based detection, classification and prediction/prognosis in medical imaging: towards radiophenomics. PET Clin. 2022;17:183–212. 34809866 10.1016/j.cpet.2021.09.010

[20] Cui J Gong K Guo N Kim K Liu H. CT-guided PET parametric image reconstruction using deep neural network without prior training data. In: *Proceedings of SPIE 10948, Medical Imaging 2019: Physics of Medical Imaging.* SPIE; 2019:109480Z.

[21] Xie N Gong K Guo N.Clinically translatable direct Patlak reconstruction from dynamic PET with motion correction using convolutional neural network. In: *Medical Image Computing and Computer Assisted Intervention: MICCAI 2020.* Springer International Publishing; 2020:793–802.

[22] Gong K Catana C Qi J Li Q. Direct reconstruction of linear parametric images from dynamic PET using nonlocal deep image prior. IEEE Trans Med Imaging. 2022;41:680–689. 10.1109/TMI.2021.3120913 PMC8956450 34652998

[23] Jackson P Hardcastle N Dawe N Kron T Hofman MS Hicks RJ. Deep learning renal segmentation for fully automated radiation dose estimation in unsealed source therapy. Front Oncol. 2018;8:215. 29963496 10.3389/fonc.2018.00215 PMC6010550

[24] Akhavanallaf A Shiri I Arabi H Zaidi H. Whole-body voxel-based internal dosimetry using deep learning. Eur J Nucl Med Mol Imaging. 2021;48:670–682. 32875430 10.1007/s00259-020-05013-4 PMC8036208

[25] Langlotz CP Allen B Erickson BJ. A roadmap for foundational research on artificial intelligence in medical imaging: from the 2018 NIH/RSNA/ACR/the Academy Workshop. Radiology. 2019;291:781–791. 30990384 10.1148/radiol.2019190613 PMC6542624

[26] Morris MA Saboury B Burkett B Gao J Siegel EL. Reinventing radiology: big data and the future of medical imaging. J Thorac Imaging. 2018;33:4–16. 29252898 10.1097/RTI.0000000000000311

[27] Sitek A Ahn S Asma E. Artificial intelligence in PET: an industry perspective. PET Clin. 2021;16:483–492. 34353746 10.1016/j.cpet.2021.06.006

[28] Krizhevsky A Sutskever I Hinton GE. ImageNet classification with deep convolutional neural networks. In: *Advances in Neural Information Processing Systems 25 (NIPS 2012)*. MIT Press; 2012:1–9.

[29] Ouyang D He B Ghorbani A. Video-based AI for beat-to-beat assessment of cardiac function. Nature. 2020;580:252–256. 32269341 10.1038/s41586-020-2145-8 PMC8979576

[30] Huang S-C Pareek A Seyyedi S Banerjee I Lungren MP. Fusion of medical imaging and electronic health records using deep learning: a systematic review and implementation guidelines. NPJ Digit Med. 2020;3:136. 33083571 10.1038/s41746-020-00341-z PMC7567861

[31] Kaissis G Ziller A Passerat-Palmbach J. End-to-end privacy preserving deep learning on multi-institutional medical imaging. Nat Mach Intell. 2021;3:473–484.

[32] Warnat-Herresthal S Schultze H Shastry KL. Swarm Learning for decentralized and confidential clinical machine learning. Nature. 2021;594:265–270. 34040261 10.1038/s41586-021-03583-3 PMC8189907

[33] Begoli E Bhattacharya T Kusnezov D. The need for uncertainty quantification in machine-assisted medical decision making. Nat Mach Intell. 2019;1:20–23.

[34] Poplin R Varadarajan AV Blumer K. Prediction of cardiovascular risk factors from retinal fundus photographs via deep learning. Nat Biomed Eng. 2018;2:158–164. 31015713 10.1038/s41551-018-0195-0

[35] Ghassemi M Oakden-Rayner L Beam AL. The false hope of current approaches to explainable artificial intelligence in health care. Lancet Digit Health. 2021;3:e745–e750. 34711379 10.1016/S2589-7500(21)00208-9

[36] Arun N Gaw N Singh P. Assessing the trustworthiness of saliency maps for localizing abnormalities in medical imaging. Radiol Artif Intell. 2021;3:e200267. 34870212 10.1148/ryai.2021200267 PMC8637231

[37] Obermeyer Z Powers B Vogeli C Mullainathan S. Dissecting racial bias in an algorithm used to manage the health of populations. Science. 2019;366:447–453. 31649194 10.1126/science.aax2342

[38] Murray E Treweek S Pope C. Normalisation process theory: a framework for developing, evaluating and implementing complex interventions. BMC Med. 2010;8:63. 20961442 10.1186/1741-7015-8-63 PMC2978112

[39] Morris ZS Wooding S Grant J. The answer is 17 years, what is the question: understanding time lags in translational research. J R Soc Med. 2011;104:510–520. 22179294 10.1258/jrsm.2011.110180 PMC3241518

[40] May C. A rational model for assessing and evaluating complex interventions in health care. BMC Health Serv Res. 2006;6:86. 16827928 10.1186/1472-6963-6-86 PMC1534030

[41] Gong K Kim K Cui J Wu D Li Q. The evolution of image reconstruction in PET: from filtered back-projection to artificial intelligence. PET Clin. 2021;16:533–542. 34537129 10.1016/j.cpet.2021.06.004

[42] McMillan AB Bradshaw TJ. Artificial intelligence–based data corrections for attenuation and scatter in position emission tomography and single-photon emission computed tomography. PET Clin. 2021;16:543–552. 34364816 10.1016/j.cpet.2021.06.010 PMC10562009

[43] Liu J Malekzadeh M Mirian N Song T-A Liu C Dutta J. Artificial intelligence-based image enhancement in PET imaging: noise reduction and resolution enhancement. PET Clin. 2021;16:553–576. 34537130 10.1016/j.cpet.2021.06.005 PMC8457531

[44] Yousefirizi F Jha AK Brosch-Lenz J Saboury B Rahmim A. Toward high-throughput artificial intelligence-based segmentation in oncological PET imaging. PET Clin. 2021;16:577–596. 34537131 10.1016/j.cpet.2021.06.001

[45] Cross DJ Komori S Minoshima S. Artificial intelligence for brain molecular imaging. PET Clin. 2022;17:57–64. 34809870 10.1016/j.cpet.2021.08.001

[46] Gharavi SMH Faghihimehr A. Clinical application of artificial intelligence in PET imaging of head and neck cancer. PET Clin. 2022;17:65–76. 34809871 10.1016/j.cpet.2021.09.004

[47] Zukotynski KA Gaudet VC Uribe CF Chiam K Bénard F Gerbaudo VH. Clinical applications of artificial intelligence in positron emission tomography of lung cancer. PET Clin. 2022;17:77–84. 34809872 10.1016/j.cpet.2021.09.001

[48] Miller RJH Singh A Dey D Slomka P. Artificial intelligence and cardiac PET/computed tomography imaging. PET Clin. 2022;17:85–94. 34809873 10.1016/j.cpet.2021.06.011

[49] Slart RHJA Williams MC Juarez-Orozco LE. Position paper of the EACVI and EANM on artificial intelligence applications in multimodality cardiovascular imaging using SPECT/CT, PET/CT, and cardiac CT. Eur J Nucl Med Mol Imaging. 2021;48:1399–1413. 33864509 10.1007/s00259-021-05341-z PMC8113178

[50] Paravastu SS Theng EH Morris MA. Artificial intelligence in vascular-PET: translational and clinical applications. PET Clin. 2022;17:95–113. 34809874 10.1016/j.cpet.2021.09.003

[51] Paravastu SS Hasani N Farhadi F. Applications of artificial intelligence in ^18^F-sodium fluoride positron emission tomography/computed tomography: current state and future directions. PET Clin. 2022;17:115–135. 34809861 10.1016/j.cpet.2021.09.012

[52] Ma K Harmon SA Klyuzhin IS Rahmim A Turkbey B. Clinical application of artificial intelligence in positron emission tomography: imaging of prostate cancer. PET Clin. 2022;17:137–143. 34809863 10.1016/j.cpet.2021.09.002

[53] Hasani N Paravastu SS Farhadi F. Artificial intelligence in lymphoma PET imaging: a scoping review (current trends and future directions). PET Clin. 2022;17:145–174. 34809864 10.1016/j.cpet.2021.09.006 PMC8735853

[54] Wang Y Li E Cherry SR Wang G. Total-body PET kinetic modeling and potential opportunities using deep learning. PET Clin. 2021;16:613–625. 34353745 10.1016/j.cpet.2021.06.009 PMC8453049

[55] Ding W Yu J Zheng C. Machine learning-based noninvasive quantification of single-imaging session dual-tracer ^18^F-FDG and ^68^Ga-DOTATATE dynamic PET-CT in oncology. IEEE Trans Med Imaging. 2022;41:347–359. 34520350 10.1109/TMI.2021.3112783

[56] Tunyasuvunakool K Adler J Wu Z. Highly accurate protein structure prediction for the human proteome. Nature. 2021;596:590–596. 34293799 10.1038/s41586-021-03828-1 PMC8387240

[57] Webb EW Scott PJH. Potential applications of artificial intelligence and machine learning in radiochemistry and radiochemical engineering. PET Clin. 2021;16:525–532. 34537128 10.1016/j.cpet.2021.06.012 PMC9168959

[58] Ataeinia B Heidari P. Artificial intelligence and the future of diagnostic and therapeutic radiopharmaceutical development: in silico smart molecular design. PET Clin. 2021;16:513–523. 34364818 10.1016/j.cpet.2021.06.008 PMC8453048

[59] Arabi H. AkhavanAllaf A, Sanaat A, Shiri I, Zaidi H. The promise of artificial intelligence and deep learning in PET and SPECT imaging. Phys Med. 2021;83:122–137. 33765602 10.1016/j.ejmp.2021.03.008

[60] Shi L Onofrey JA Liu H Liu Y-H Liu C. Deep learning-based attenuation map generation for myocardial perfusion SPECT. Eur J Nucl Med Mol Imaging. 2020;47:2383–2395. 32219492 10.1007/s00259-020-04746-6

[61] Brosch-Lenz J Yousefirizi F Zukotynski K. Role of artificial intelligence in theranostics: toward routine personalized radiopharmaceutical therapies. PET Clin. 2021;16:627–641. 34537133 10.1016/j.cpet.2021.06.002

[62] Beegle C Hasani N Maass-Moreno R Saboury B Siegel E. Artificial intelligence and positron emission tomography imaging workflow. PET Clin. 2022;17:31–39. 34809867 10.1016/j.cpet.2021.09.008 PMC8797670

[63] Ullah MN Levin CS. Application of artificial intelligence in PET instrumentation. PET Clin. 2022;17:175–182. 34809865 10.1016/j.cpet.2021.09.011

[64] Yang J Sohn JH Behr SC Gullberg GT Seo Y. CT-less direct correction of attenuation and scatter in the image space using deep learning for whole-body FDG PET: potential benefits and pitfalls. Radiol Artif Intell. 2020;3:e200137. 33937860 10.1148/ryai.2020200137 PMC8043359

[65] Mezrich JL. Demystifying medico-legal challenges of artificial intelligence applications in molecular imaging and therapy. PET Clin. 2022;17:41–49. 34809868 10.1016/j.cpet.2021.08.002

[66] Saboury B Morris M Siegel E. Future directions in artificial intelligence. Radiol Clin North Am. 2021;59:1085–1095. 34689876 10.1016/j.rcl.2021.07.008

[67] Yousefi Nooraie R Lyons PG Baumann AA Saboury B. Equitable implementation of artificial intelligence in medical imaging: what can be learned from implementation science? PET Clin. 2021;16:643–653. 34537134 10.1016/j.cpet.2021.07.002 PMC12121049

[68] Martinho A Kroesen M Chorus C. A healthy debate: exploring the views of medical doctors on the ethics of artificial intelligence. Artif Intell Med. 2021;121:102190. 34763805 10.1016/j.artmed.2021.102190

[69] Hasani N Morris MA Rhamim A. Trustworthy artificial intelligence in medical imaging. PET Clin. 2022;17:1–12. 34809860 10.1016/j.cpet.2021.09.007 PMC8785402

[70] Kilbride MK Joffe S. The new age of patient autonomy: implications for the patient-physician relationship. JAMA. 2018;320:1973–1974. 30326026 10.1001/jama.2018.14382 PMC6988779

[71] Lyell D Coiera E. Automation bias and verification complexity: a systematic review. J Am Med Inform Assoc. 2017;24:423–431. 27516495 10.1093/jamia/ocw105 PMC7651899

[72] Vinuesa R Azizpour H Leite I. The role of artificial intelligence in achieving the sustainable development goals. Nat Commun. 2020;11:233. 31932590 10.1038/s41467-019-14108-y PMC6957485

[73] Jha AK Myers KJ Obuchowski NA. Objective task-based evaluation of artificial intelligence-based medical imaging methods: framework, strategies, and role of the physician. PET Clin. 2021;16:493–511. 34537127 10.1016/j.cpet.2021.06.013

[74] Grant ES. Requirements engineering for safety critical systems: an approach for avionic systems. In: *2016 2nd IEEE International Conference on Computer and Communications (ICCC).* IEEE; 2016:991–995.

[75] Zhou Q Zuley M Guo Y. A machine and human reader study on AI diagnosis model safety under attacks of adversarial images. Nat Commun. 2021;12:7281. 34907229 10.1038/s41467-021-27577-x PMC8671500

[76] Hasani N Farhadi F Morris MA. Artificial intelligence in medical imaging and its impact on the rare disease community: threats, challenges and opportunities. PET Clin. 2022;17:13–29. 34809862 10.1016/j.cpet.2021.09.009 PMC8764708

[77] Rudin C. Stop explaining black box machine learning models for high stakes decisions and use interpretable models instead. Nat Mach Intell. 2019;1:206–215. 35603010 10.1038/s42256-019-0048-x PMC9122117

[78] Harvey DL. Agency and community: a critical realist paradigm. J Theory Soc Behav. 2002;32:163–194.

[79] Science and Technology Policy Office. Notice of request for information (RFI) on public and private sector uses of biometric technologies. Fed Regist. 2021;86:56300–56302.

